# Neoadjuvant combination treatment with checkpoint inhibitors, chemotherapy, and BRAF/MEK inhibitors for BRAF^V600E^ glioblastoma results in sustained response: A case report

**DOI:** 10.1093/noajnl/vdae110

**Published:** 2024-07-02

**Authors:** Naveed Wagle, Akanksha Sharma, Minhdan Nguyen, Judy Truong, Tiffany M Juarez, Santosh Kesari

**Affiliations:** Pacific Neuroscience Institute, Saint John’s Cancer Institute, Providence Saint John’s Health Center, Saint Monica, California, USA; Pacific Neuroscience Institute, Saint John’s Cancer Institute, Providence Saint John’s Health Center, Saint Monica, California, USA; Pacific Neuroscience Institute, Saint John’s Cancer Institute, Providence Saint John’s Health Center, Saint Monica, California, USA; Pacific Neuroscience Institute, Saint John’s Cancer Institute, Providence Saint John’s Health Center, Saint Monica, California, USA; CureScience Institute, San Diego, California, USA; Pacific Neuroscience Institute, Saint John’s Cancer Institute, Providence Saint John’s Health Center, Saint Monica, California, USA

**Keywords:** BRAFV600E, case report, checkpoint inhibitors, glioblastoma, neoadjuvant

## Abstract

Radiation’s confounding and adverse effects on tumor microenvironment and normal brain could potentially be delayed by upfront combination treatment. We present a patient with newly diagnosed *BRAF*^V600E^-mutant, PD-L1-positive glioblastoma treated with off-label RAF/MEK inhibitors encorafenib/binimetinib after progressing on postoperative immune checkpoint blockade and temozolomide (no radiation administered: NCT03425292). Complete response occurred 6 months after adding encorafenib/binimetinib, and clinical benefit was sustained for over 20 months. Treatment was well tolerated with manageable toxicities, with quality of life and cognitive function maintained throughout treatment. Adding encorafenib/binimetinib to immunotherapy and temozolomide conferred favorable and lasting efficacy for our *BRAF*^*V600E*^-mutant glioblastoma patient, justifying future studies.

**Glioblastoma (GBM) is the most common and aggressive primary brain tumor with uniformly poor outcomes despite standard upfront chemoradiation with temozolomide and tumor-treating fields.^1^ Median survival remains under 2 years and more effective treatments are needed. Despite the failure of immune checkpoint blockade (ICB) to improve survival for GBM patients in the adjuvant setting (CheckMate-143,^2^ CheckMate-498^3^), subgroup analyses of clinical trials suggest there are underlying characteristics of brain cancers that may influence responsiveness to immunotherapy.^4–6^ Success of ICB in several cancer types and the potential to target malignant cells while sparing normal tissue remain attractive features for continued investigation in GBM. In addition to overcoming the inherent immunosuppressive microenvironment in GBM, the timing of immunotherapy along the treatment course may be an important consideration for glioma susceptibility to immunotherapy, as evidenced by antitumor responses seen in recurrent GBM treated with presurgical neoadjuvant anti-PD-1 therapy.^7,8^ Furthermore, the addition of chemotherapy or targeted agents to ICB has led to FDA approval in other solid tumors such as nivolumab (anti-PD-1) plus platinum-doublet chemotherapy for the neoadjuvant treatment of patients with resectable non–small cell lung cancer^9^ and pembrolizumab (anti-PD-1) plus lenvatinib (VEGFR kinase inhibiter) in renal cell carcinoma.^10^**

We are currently evaluating ICB combined with chemotherapy and targeted agents when actionable tumor mutations are present. Many tumor types have alterations in the mitogen-activated protein kinase (MAPK) pathway that lead to constitutive activation of downstream signaling that promotes cell proliferation and survival. Inhibitors of BRAF and MEK in the MAPK pathway have been shown to have survival benefits for patients with *BRAF*^V600E^-mutant neoplasms including advanced melanoma and recurrent gliomas.^11–15^ Although *BRAFV600E* mutations are found in less than 3% of IDH-wild-type glioblastomas, selective targeting of the *BRAFV600E* oncogene represents a strategy for potentially improving outcomes.^16^ We describe our experience using combined targeted therapy in the neoadjuvant setting for a patient with *BRAF*^V600E^-mutant glioblastoma followed by the addition of BRAF and MEK inhibitors (Figure 1A).

**Figure 1.**
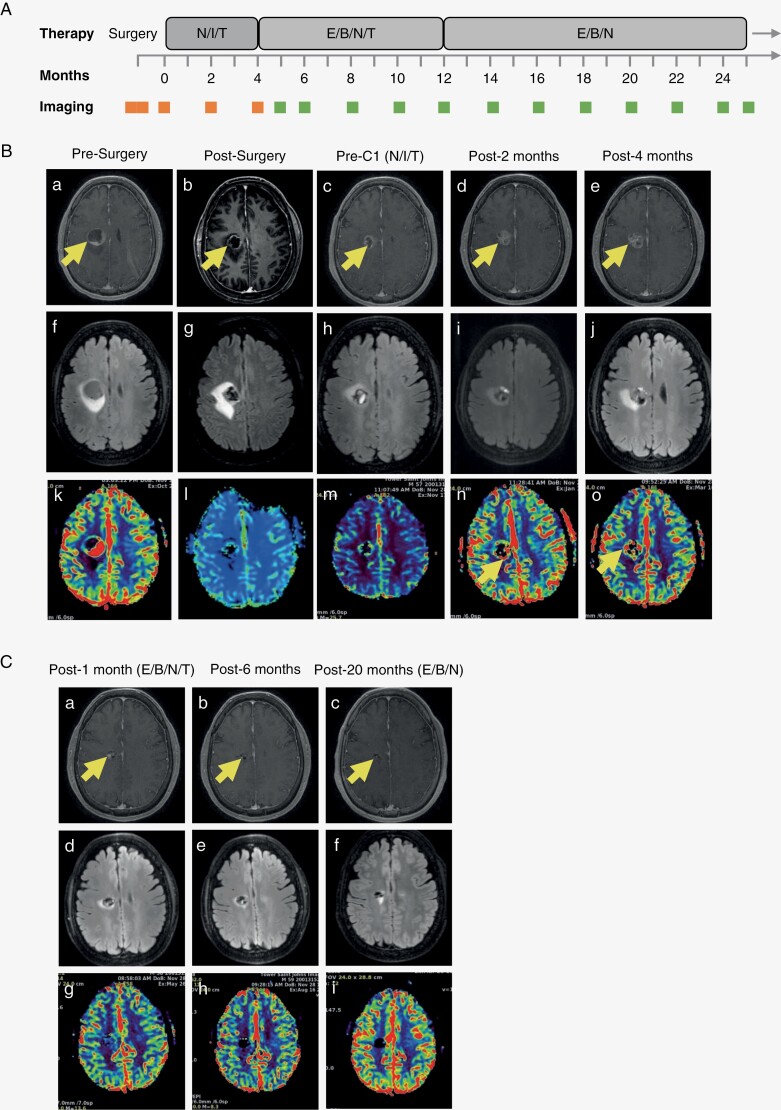
Treatment protocol and radiological findings. (A) Timeline and treatment protocol, with rectangles indicating times of brain scans. The patient was treated with nivolumab 240 mg every 2 weeks, ipilimumab 1 m/kg every 6 weeks, and temozolomide (N/I/T) and followed with monthly scans. At progression, ipilimumab was discontinued, and encorafenib and binimetinib were added to the treatment regimen and (E/B/N/T). Temozolomide was discontinued after six cycles combined with E/B/N. (B). T1 postcontrast images (a–e), edema on axial FLAIR sequences (f–j), and axial T1 perfusion sequences (k–o) over time from initial surgery to progression on N/I/T. (C) T1 postcontrast (a–c), edema on axial FLAIR sequences (d–e) and axial T1 perfusion sequences (g–i) with E/B/N/T.

A male in his 50s with no significant medical history presented with left-sided weakness and headaches. MRI brain showed a 3.0 × 3.0 × 2.5 cm^3^ cystic right frontal mass with cerebral edema, mass effect, and elevated perfusion consistent with primary high-grade glioma (Figure 1B, a, f, k). The patient underwent a craniotomy with near gross total resection on MRI within 48 hours following surgery (Figure 1B, b, g, l) but pretreatment baseline MRI 4 weeks later already showed growth around the resection cavity (Figure 1B, c, h, m). Pathology showed a highly cellular, pleomorphic tumor with necrosis, prominent vascular proliferation, and a Ki67 index of 45%. Molecular profiling of the tumor using CARIS MI panel revealed methylated MGMT promoter, wild-type P53/IDH1/IDH2, intact PTEN/ATRX, MSI stable, TMB low, *BRAF*^V600E^ mutation, and positive expression of programmed cell death protein 1 (PD-1) and programmed death-ligand 1 (PD-L1) (60%) by immunohistochemistry with WHO 2021 diagnosis of glioblastoma. The patient expressed interest in treatment options that did not include radiation even though it was explained to him that radiotherapy was the current standard of care. He was offered participation in our clinical trial of preradiation (“neoadjuvant”) temozolomide plus nivolumab (anti-PD-1) and ipilimumab (cytotoxic T-lymphocyte antigen 4 [CTLA-4] inhibitor) (NCT03425292). He received treatment on study; however, the tumor progressed after 4 months on MRI in the absence of clinical progression (Figure 1B, d–e, i–j, n–o). The imaging changes were felt to be progressive disease based on the increase in perfusion over time (Figure 1B, m–o), although we cannot fully exclude a component of pseudoprogression. Pseudoprogression occurs more in patients whose gliomas are methylated and described in the setting of chemoradiation as well as in brain metastases that receive high doses of radiation.^17–20^ However, this may or may not apply without radiation and certainly does not seem to occur in naïve brain metastases that receive checkpoint inhibitors.^21,22^

The patient again expressed interest in treatment options that did not include radiation. Ipilimumab was discontinued, and based on the *BRAF*^V600E^ mutation, encorafenib (BRAF inhibitor) and binimetinib (MEK inhibitor) were added off-label while continuing temozolomide and nivolumab. The combination was tolerated well without any additional side effects. Imaging 1 month later showed rapid partial response and reduction in edema without any steroids (Figure 1C, a, d, g), and complete response was documented at 6 months of treatment (Figure 1C, b, e, h). The patient continued temozolomide for a total of 12 cycles and continued encorafenib, binimetinib, and nivolumab for over 20 months without progression (Figure 1C, c, f, i). The patient continues to have a great quality of life and has continued to work full time throughout treatment without the significant cognitive and systemic side effects that are prevalent with upfront chemoradiation.

Here we present a case of a patient with BRAFV600E and PD-1-positive GBM who received novel preradiation of chemotherapy and immune checkpoint inhibitors, followed by the addition of RAF/MEK-targeted therapy after initial progression. This treatment regimen has enabled the patient to defer radiation for over 24 months and maintain a high quality of life. In the phase 3 CATNON study of temozolomide and radiotherapy versus radiotherapy alone in newly diagnosed IDH-wild-type glioblastoma, post-hoc analysis revealed patients with *MGMT*-methylated tumors had superior overall survival compared to *MGMT*-unmethylated tumors (HR 0.65; 95% CI 0.45–0.92), with a median overall survival of 1.8 years versus 1.4 years, respectively.^23^ The delay of radiation did not appear to negatively impact the survival of our patient, although a larger sample size is certainly required.

Given the patient was on combination therapy, some of the response and benefit may be driven by temozolomide even though the tumor initially progressed on the combination of temozolomide, nivolumab, and ipilimumab. Likewise, considering the melanoma DREAMseq study (NCT02224781), early exposure to nivolumab and ipilimumab might bestow a benefit to subsequent treatment with RAF/MEK-targeted therapy. Nevertheless, this case report highlights the need to develop novel therapeutic combinations of upfront personalized therapy based on molecular profiling in a clinical trial setting.

A recent report of encorafenib and binimetinib administered to 5 patients with recurrent *BRAF*^*V600E*^-mutated high-grade glioma revealed a complete response in 2 out of 2 patients with glioblastoma.^24^ Although the trial was stopped due to enrollment issues, the promising tumor responses add to the growing body of support for targeting *BRAF*^*V600E*^ in glioblastoma.

This case supports the use of BRAF and MEK inhibition for the treatment of *BRAF*^*V600E*^ glioblastoma in the upfront setting. An additional consideration needing further evaluation is administering such treatment prior to standard radiation. Upfront neoadjuvant precision medicine approaches have the potential to make significant improvements in glioblastoma survival while maintaining optimal cognitive function in selected patients and should be explored more in the current era of advanced targeted therapies and immunotherapies. It is increasingly clear that a combination of checkpoint inhibitors with targeted therapies or chemotherapies in certain cases can significantly improve response and outcomes.^25,26^ While difficult to tease apart the individual contributions of each drug in this case, the clear objective response and durability suggest that neoadjuvant combination treatments in a clinical trial setting of a heterogeneous disease such as glioblastoma are warranted.
